# Compact hematite buffer layer as a promoter of nanorod photoanode performances

**DOI:** 10.1038/srep35049

**Published:** 2016-10-13

**Authors:** R. Milan, S. Cattarin, N. Comisso, C. Baratto, K. Kaunisto, N. V. Tkachenko, I. Concina

**Affiliations:** 1 Department of Information Engineering, University of Brescia, Via Branze 38, 9–25131 Brescia, Italy; 2 CNR-INO SENSOR Laboratory, via Branze 45–25123 Brescia, Italy; 3 ICMATE–CNR, Corso Stati Uniti 4, 35127 Padova, Italy; 4 Department of Chemistry and Bioengineering, Tampere University of Technology, P.O. Box 541, FI-33101 Tampere, Finland; 5 Division of Materials Science, Department of Engineering Sciences and Mathematics, Luleå University of Technology, 971 87 Luleå, Sweden

## Abstract

The effect of a thin α-Fe_2_O_3_ compact buffer layer (BL) on the photoelectrochemical performances of a bare α-Fe_2_O_3_ nanorods photoanode is investigated. The BL is prepared through a simple spray deposition onto a fluorine-doped tin oxide (FTO) conducting glass substrate before the growth of a α-Fe_2_O_3_ nanorods via a hydrothermal process. Insertion of the hematite BL between the FTO and the nanorods markedly enhances the generated photocurrent, by limiting undesired losses of photogenerated charges at the FTO||electrolyte interface. The proposed approach warrants a marked improvement of material performances, with no additional thermal treatment and no use/dispersion of rare or toxic species, in agreement with the principles of green chemistry.

Bard and Hardee^1^ demonstrated some decades ago the potential hold by hematite (α-phase of iron oxide) as a suitable material for photoelectrochemical water oxidation. Since that pioneering work the interest on hematite has kept growing and this material is possibly the most studied n-type semiconductor in the field of photoelectrochemical water splitting to date, i.e. for the generation of clean chemical fuels (oxygen and hydrogen) through the breaking of water molecules. This interest is especially due to some critical advantages retained by hematite, including rather good light absorption, extreme stability in harsh aqueous environment, abundance of its components, low/no significant toxicity and the remarkable potential to convert up to 16.8% of solar energy into hydrogen^2^. At the same time, relevant drawbacks have been highlighted during the time: poor optoelectronic properties (leading to low light harvesting efficiency and large required overpotential for water oxidation), short minority carrier lifetime and diffusion length (2–3 nm)^1^,^3^,^4^,^5^,^6^, relatively sluggish kinetics of surface water oxidation. However, these limitations have not restricted the interest in applying hematite as anode for water splitting and efforts are devoted to improve the capability of these electrodes in oxygen generation. A recent review on the topic^7^ has highlighted how an approach of engineering the structure of either hematite or the electrode can significantly increase the overall performances of α-Fe_2_O_3_. High charge carrier recombination in hematite is one of the critical problems to face. To reduce the recombination rate of photogenerated charges, the use of quasi 1D structures, such as nanorods or nanowires, has been proposed. This approach would favor the holes to reach the oxide||electrolyte interface, oxidizing the water before the recombination takes place^8^,^9^. In this frame, Vayssieres and coworkers proposed the inexpensive fabrication of large scale area of vertically aligned iron oxide nanorods (NRs), directly grown on a polycrystalline substrate by means of a hydrothermal process^10^. Through these structures photogenerated charges have in principle a preferential path to the back contact, since no grain boundaries affect the charge transport^11^. Although the structure of NRs is suitable for this application, electrode performances suffer from losses due to bulk recombination and electron back-injection into the electrolyte on the exposed areas of FTO^12^,^13^, whose reduction has been subject of several investigations, especially focusing on improving the charge transport through either thermal treatment or doping. Thermal treatments at temperature higher than 700 °C affect the original material nature: high degree of defects, alteration of the preferential orientation of the crystals and polycrystallinity were indeed observed with transmission electronic microscopy^14^, which alter the overall efficiency of electron transport process, resulting in an enhancement of the exciton recombination rate. Nonetheless, the net effect is an enhancement of the photogenerated current, possibly ascribable to tin (IV) self-doping from the FTO^15^,^16^.

Doping of hematite NRs with different materials, such as Mn^17^, Pt^18^,^19^, Ta^20^, graphene and BiV_1-x_Mo_x_O_4_^21^ may also markedly enhance the photogenerated current.

It has been also demonstrated that the deposition of dense underlayers of SiO_x_, TiO_2_, Nb_2_O_5_, or Ga_2_O_3_ between the FTO and the metal oxide semiconductor increases the delivered photocurrent^13^,^22^, although the actual mechanism behind this is not yet clarified. Introduction of underlayers and subsequent annealing of the layered electrode could induce morphological changes^22^, as well as temperature-driven ionic diffusion doping of hematite with species present in the underlayer^23^,^24^. Recently Cho *et al*.^25^ proposed to use thermal treatment, Ti-doping and use of Fe_2_O_3_:Ti underlayer to simultaneously reduce bulk, interface and surface recombination phenomena, without morphology variation of the NRs and limited self-diffusion doping^26^. Annamalai^27^ proposed in the same year the use of a TiO_2_ blocking layer to reduce the recombination of charges and a thermal treatment for enhancing the photoconversion rate (resulting again in self doping of hematite).

In the present investigation, a simple electrode modification is proposed, consisting of the formation of a thin compact hematite buffer layer (BL) over the FTO substrate, inhibiting the undesired losses of charges at the FTO||electrolyte interface. The hematite BL is inserted in between the FTO and the active α-Fe_2_O_3_ layer to physically insulate the FTO from the electrolyte, hence exploiting a strategy already proved highly beneficial in electrodes for excitonic solar cells^28^,^29^ and expected to drastically reduce the contribution of the FTO substrate to reactions with the electrolyte. With this strategy, neither thermal nor doping treatment are involved. The use of hematite as BL eliminates any chance of self-doping, thus ensuring that observed performance enhancement is exclusively due to the reduction of charge losses at the FTO||electrolyte interface, which is a critical step in view of the fabrication of efficient photoelectrodes for PECs.

## Results and Discussion

**Figure 1:**
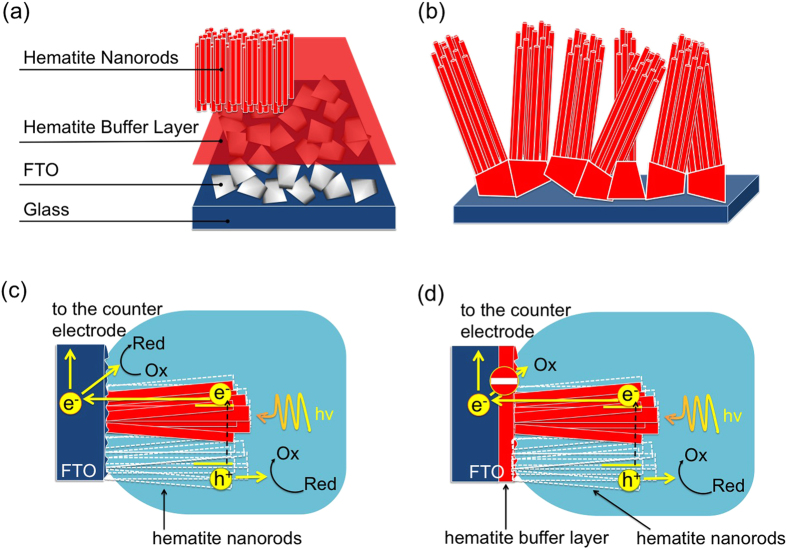
Scheme illustrating the concept of insulation of FTO using a very thin layer of hematite. (**a**) Architecture of the electrode showing each component; (**b**) hematite NRs vertically grown on the substrate provided with the hematite blocking layer, showing inclination with respect to the vertical due to FTO morphology. Processes occurring during electrode irradiation: back reduction processes occur at the FTO||electrolyte interface (**c**) but are blocked in the presence of an interposed BL (**d**).

Figure 1 illustrates the concept applied in the present work: layered electrodes are fabricated (Fig. 1a), in which a thin hematite layer is generated by spray in between the FTO and the hematite NRs, grown conformally by *in situ* hydrothermal synthesis (Fig. 1b). Presence of hematite BL is then expected to enhance the photocurrent generation efficiency by reducing the charge carrier losses at the interface FTO||electrolyte (Fig. 1c,d).

**Figure 2:**
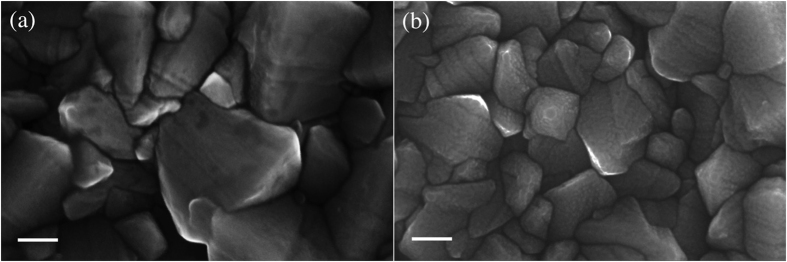
SEM images of (**a**) bare FTO glass and (**b**) FTO glass covered by hematite BL. Scale bar: 100 nm.

Hematite NRs are expected to present some deviation from a perfect vertical alignment with respect to the glass substrate (as schematically depicted in Fig. 1b) due to the shape of FTO crystallites and thinness of the hematite BL atop (see electron microscopy analysis reported in Fig. 2) as well as a reduced compactness in correspondence with FTO underlying grains.

Figure 2 shows the SEM comparison of a bare FTO glass and a FTO glass covered by the thin hematite BL, resulting in homogeneously deposited hematite layer on the substrate. It is worth noting that FTO appearance is not modified by the spray deposition of hematite BL, which is constituted by very small particles (visible in the high magnification image reported in Fig. 2b, whose size are evaluated smaller than 20 nm).

**Figure 3:**
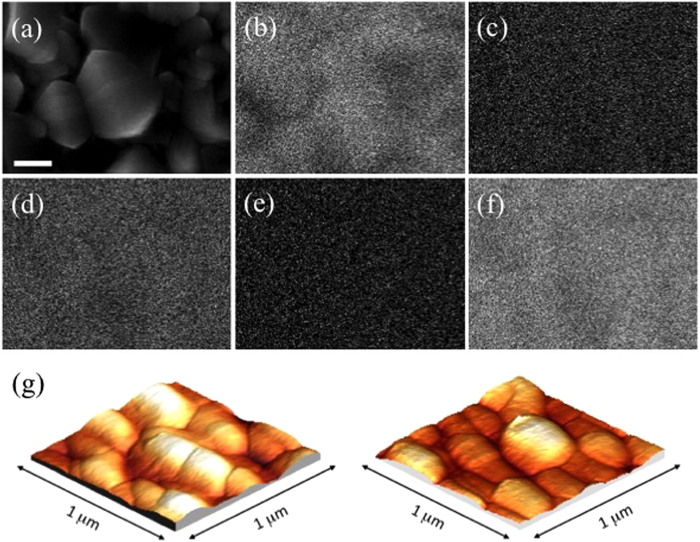
(**a–f**): EDX analysis of hematite BL spray deposited on FTO glass. (**a**): SEM image of selected area; (**b**) O, (**c**) Si, (**d**) Ca, (**e**) Fe, (**f**) Sn. Scale bar: 200 nm. (**g**) AFM analysis of FTO glass prior (left) and after (right) hematite BL deposition.

EDX elemental maps (Fig. 3a–f) confirm the complete conformal coverage of the substrate by hematite BL, detecting iron homogeneously distributed on FTO crystallites. Elements pertaining to both FTO and glass are also identified, confirming the formation of a thin film. Substrate roughness is slightly increased by the deposition of hematite BL, as evidenced by AFM investigation (reported in Fig. 3g), through which values of 19 and 23 of root mean square surface roughness are measured for the bare FTO glass and for the FTO glass with hematite BL, respectively.

**Figure 4:**
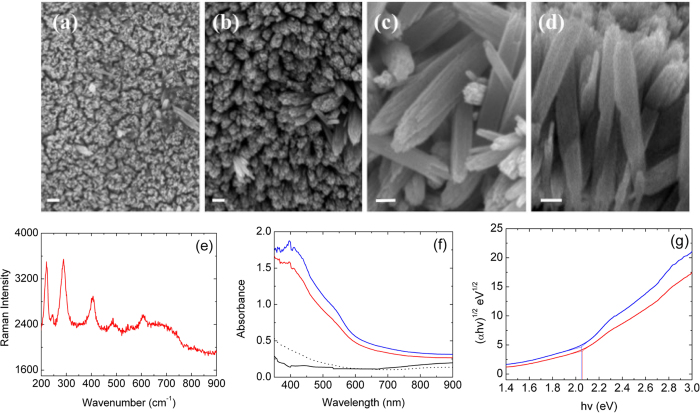
(**a–d**) SEM images of hematite nanorods grown on FTO glass after hematite BL deposition. Scale bars: 200 nm (**a**) and 100 nm (**b–d**). (**e**) Raman spectrum of NRs@BL-FTO glass. (**f**) Absorption spectra of samples under investigation (recorded in back irradiation geometry) and Tauc plots for (**g**) indirect allowed transitions: FTO (black solid line); FTO covered with hematite BL (black dotted line); hematite NRs (red line); hematite NRs deposited on hematite BL (blue line).

Hydrothermal reaction results in the deposition of hematite NRs reasonably perpendicular to the substrate (Fig. 4a) and constituting a homogeneous layer. Slight inclination with respect to the vertical can be appreciated (Fig. 4b), as a result of the uneven morphology of the underlying FTO layer, as shown in the scheme of Fig. 1. Higher magnification SEM imaging reveals that individual rods are composed by smaller wires clustered together (Fig. 4c,d). The NRs feature a length of about 500 nm and diameter close to 50 nm (single wires size less than 10 nm).

Raman analysis (Fig. 4e) confirmed the formation of hematite as the unique crystal phase of iron oxide. Raman modes characterizing the α crystalline form of iron oxide are visible: peaks centered at 222 and 496 cm^−1^ pertain to A_1g_ mode, while signals at 242, 291, 408 and 610 cm^−1^ are attributed to E_g_ mode. A broad band is visible in the region from 600 to 700 cm^−1^: in this interval peaks pertaining to magnetite (667 cm^−1^) and maghemite (671 cm^−1^) phases may be found^30^, which would however be narrower and better defined than in our case.

In previous literature many hematite samples have been found to show a peak in the region between 600 and 700 cm^−1^ (usually centered at 660 cm^−1^). Sivula *et al*.^31^ observed for instance a peak at 660 cm^−1^ in thin films of hematite that was initially attributed to the presence of magnetite phase, but later excluded by Mossbauer analysis. Bersani *et al*.^32^ investigated hematite thin films by means of micro Raman, highlighting the presence of the peak adducible in the first instance to the magnetite. They verified that the needed hematite/magnetite ratio to make the peak visible is 1/1 and this finding spurred them to abandon the hypothesis of magnetite presence. They concluded that the presence of the peak at 660 cm^−1^ is possibly due to the disorder-induced activation of the longitudinal optical E_u_ IR-active mode present at the same wavenumber^33^. This hypothesis has been subsequently confirmed by independent investigations^34^,^35^,^36^ and in the present paper we conform to this explanation.

Figure 4f reports the absorption spectra of the samples under investigation. FTO shows a very good transparency over the whole UV-VIS-NIR range, as expected. Spectrum of hematite BL deposited on FTO (dotted black line) shows very similar features as FTO in the range 1100–600 nm, while an increase in absorbance is detected for wavelength lower than 600 nm, ascribable to the presence of the ultra thin hematite layer. Thickness of hematite BL has been estimated around 16 nm from absorption spectra (in good agreement with SEM results), by considering a lambertian behavior and applying the Lambert-Beer law, using as absorption coefficient the value of (44 nm)^−1^ previously reported in literature at 400 nm^37^,^38^. A hematite layer featuring such a thickness does not contribute to photoelectroactivity, due to enhanced charge recombination within the layer itself promoted by interaction with the electronic cloud of the underlying fluorine-doped tin oxide^39^. Such a layer is thus expected to physically separate the photoelectro-active hematite layer from the FTO, without affecting the functional performances.

Hydrothermal growth of the hematite nanorods layer on BL results in a remarkable enhancement of absorption (red line). Optical band gap was calculated by Tauc plot (reported in Fig. 4g). Hematite has an indirect band gap^40^, although direct band gap has also been identified in case of hematite nanorods^41^. Band gap estimation from the Tauc plot considering indirect allowed transitions provides a value of 2.05 eV (samples with and without BL do not show significant differences in optical band gap), in good agreement with the evaluation reported by Beerman^41^.

The photoelectrochemical response is investigated in alkaline media. The basic photoeffects-negative photopotentials at open circuit and anodic photocurrents under polarization at sufficiently positive potentials–are those expected for an n-type semiconductor^42^,^43^.

**Figure 5:**
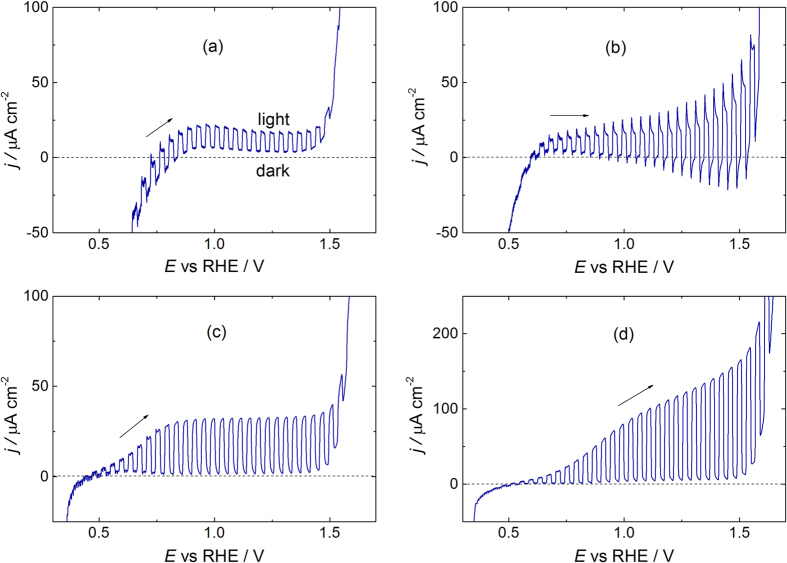
Voltammograms (scan rate 20 mV s^−1^) under chopped white light (Xe lamp, power ≅ 1 sun). (Top): 1 M NaOH, electrodes: (**a**) FTO/Hematite-NRs; (**b**) FTO/hematite-BL/hematite-NRs. (Bottom): 0.1 M NaOH + 0.2 M LiI, electrodes: (**c**) FTO/Hematite-NRs; (**d**) FTO/Hematite-BL/Hematite-NRs.

Figure 5a,b report the (photo)current-voltage (*j*_ph_-E) curves of FTO/hematite-NRs and FTO/hematite-BL/hematite-NRs photoanodes, respectively, recorded in 1 M NaOH. The response of Fig. 5a is obtained at a layer of hematite NRs grown directly on the FTO substrate. The photocurrent transients recorded during the potential scan are regular, with a well formed rectangular shape; overshoots or relaxations are minimum in the potential range of low dark current. However, the observed photocurrent is rather low, indicating large electron-hole recombination in the material.

Considering now the *j*_ph_-E curve reported in Fig. 5b, pertaining to an electrode FTO/hematite-BL/hematite-NRs, we observe a current/voltage curve somewhat stretched as compared to Fig. 5a, suggesting some hindrance of both reduction and oxidation processes^44^. Photocurrent response appears again low at negative potentials but increases markedly on sweeping the potential to positive values. Similar phenomena are observed in disordered films with a large number of localized states in the bandgap, including iron oxide films on passive iron, and indicate field-assisted photocurrent generation^45^. The shape of photocurrent transients is characterized by the presence of overshoots and decays, a response typical of systems with recombination at surface states^46^. Upon switching the light *on*, the current jumps to a large value but soon decays towards a (lower) steady state value defined by the relative rates of charge recombination and transfer to redox species. At the successive light interruption (*off* state), an overshoot of opposite sign occurs, that is associated to the filling of surface states emptied during illumination. This transient decays with a law similar to that observed for the *on* period. The shift towards negative potentials of the cathodic current (as compared to Fig. 5a) may be attributed to the suppression of the process of electron injection into solution from FTO, insulated by the hematite BL^22^.

If the same *j*_ph_-E experiments discussed in Fig. 5a,b are performed in a 1 M NaOH + 0.1 M LiI solution, the obtained pattern (not shown) is basically the same, with only an appreciable increase of the photocurrent transients in some experiments. Apparently, iodide concentrations much below those of OH^−^ do not affect appreciably the results. Conversely, in a solution of 0.1 M NaOH + 0.2 M LiI (iodide concentration larger than that of OH^−^, henceforth “iodide rich”) we obtain for both types of electrodes a marked increase of the photocurrent (Fig. 5c,d).

The curve recorded in the iodide rich solution pertaining to the electrode featuring the structure FTO/hematite-NRs (Fig. 5c) shows a markedly enhanced photoresponse as compared to that shown in Fig. 5a (no iodide). Finally, the *j*_ph_-E curve recorded for the FTO/hematite-BL/hematite-NRs (Fig. 5d) shows the best photoresponse among the explored conditions: intense rectangular transients, without the overshoots observed in the absence of iodide (Fig. 5b).

**Figure 6:**
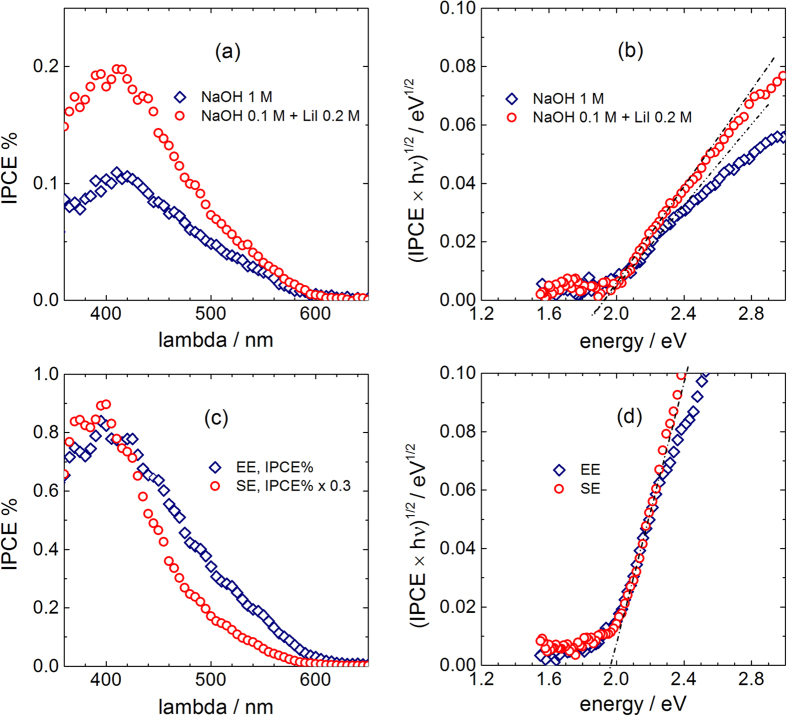
(Top): (**a**) Photocurrent action spectra of an FTO/Hematite NRs photoelectrode, recorded in the indicated media at E = 1.23 V vs RHE. Indirect transition plots in (**b**) show the same bandgap energy E_g_, slightly below 2.0 eV. (Bottom): (**c**) Photocurrent action spectra of an FTO/hematite-BL/hematite-NRs photoelectrode, recorded in 0.1 M NaOH + 0.2 M LiI at E = 1.23 V vs RHE, under front illumination from the electrolyte (EE) and back illumination from the substrate (SE). The indirect transition plots in d) allow bandgap estimation, E_g_ ≅ 1.97 eV.

Photocurrent action spectra were recorded first for a sample without BL, at a potential E = 1.23 V, where the transients are regular, both in iodide-free (1 M NaOH) and iodide-rich solutions (0.1 M NaOH + 0.2 M LiI), Fig. 6a. The IPCE plots have similar shapes, with a maximum response at about 400 nm, and a progressive decrease at longer wavelengths down to zero around 600–610 nm. However, the maximum photocurrent shows larger (about double) intensity in the iodide-rich solution. The plots according to an indirect transition law^47^ (Fig. 6b) show in both cases roughly the same intercept, slightly below 2 eV, representing an estimation of hematite bandgap in close agreement with literature^11^,^31^,^48^ and with the value obtained by absorption spectra reported in Fig. 3.

Further photospectra were recorded at electrodes with BL in 0.1 M NaOH + 0.2 M LiI (Fig. 6c), illuminating the electrode from the electrolyte side (EE) and from the substrate side (SE), in analogy to the experiments performed by Grätzel and collaborators^11^. The IPCE values are in general much larger than those obtained for the electrodes without BL (compare with Fig. 6a). Moreover, the maximum at about 400 nm (Fig. 6c) is more than 3 times larger for SE experiments than for EE experiments. If the spectra are roughly normalized, an apparent response reduction appears for SE illumination at wavelengths close to the onset, suggesting an increase of the “effective” bandgap (Fig. 6c). However, indirect transition plots of the real IPCE values (Fig. 6d) show that the bandgap is actually the same, close to 2.0 eV. Hence, the real phenomenon observed in SE experiments is a relative increase of the IPCE for high energy photons, that in these experiments are absorbed close to the substrate and generate carriers close to the point of collection, reducing losses.

The main observations are in qualitative agreement with early findings of Grätzel and collaborators on nanocrystalline hematite films^11^: rather limited IPCE values are obtained with all the illumination geometries; comparatively higher IPCE is observed for SE illumination; a marked increase of photocurrent is observed in the presence of a I^−^ concentration exceeding the concentration of OH^−^, whereas a lower relative concentration has negligible effects. We can conclude, along the same lines of the mentioned paper, that: a significant concentration of iodide promotes the photocurrent, presumably acting as a hole scavenger with the role of reducing recombination at surface states; the effect of iodide is suppressed by a much larger concentration of OH^−^, competing for adsorption on the same surface sites. It should be however remarked that for the hematite nanocrystalline films of the mentioned literature investigations, the IPCEs recorded under EE irradiation geometry were 2 orders of magnitude smaller than those observed under SE irradiation geometry, on which basis the Authors concluded that photogenerated charge collection through the nanocrystallites was particularly inefficient^11^. This is not surprising taking into account not only the mentioned intrinsic limitations in charge management featured by hematite, but also the fact that, in polycrystalline thin films, grain boundaries heavily affect the charge transport. Our results indicate that IPCEs recorded under EE irradiation are only 3 times smaller than those recorded in SE geometry.

A similar result is described in the work by Beerman *et al*.^41^, who recorded the IPCE spectra of hematite nanorod-based electrodes and found this ratio in the range 2–4, which states a significant improvement in photogenerated charge management featured by NRs and ascribable to the presence of favored path for electron transport along the quasi 1D structure. In the mentioned study, the authors used as electrolyte a 0.1M KI solution, where no hydroxyl ions were put in competition for adsorption on critical reaction sites with iodide. It is worth noting here that our results were obtained with a concentration ratio (OH^−^/I^−^) around 1/2, i.e. with an important presence of OH^−^ ions. This implies that after exciton generation, electrons are effectively drawn to the back contact along the NRs, with a remarkable positive effect exerted by the hematite BL in limiting the charge losses at the FTO||electrolyte interface.

The higher photocurrent yield obtained for back side illumination (SE) with photons of wavelength λ < 550 nm (hν > 2.25 eV) indicates that charge carriers produced close to the back contact are collected comparatively more efficiently. Conversely, photons of wavelength close to the photocurrent onset (λ > 550 nm), absorbed deep in the film “bulk”, show similar photoresponse for EE and SE illumination, as shown in Fig. 6d.

Transient absorption spectroscopy has been recently adopted as a powerful tool to study and understand charge transfer processes taking place in metal oxide photoelectrodes upon photoexcitation^49^,^50^,^51^. TAS in picosecond-to-nanosecond timescales has been carried out in order to study charge carrier dynamics. This analysis method is a relatively recent field of investigation and within the past five years several scientific reports have appeared, which helped to understand better the fate of carriers after photoexcitation^49^,^50^,^51^,^52^,^53^. In particular, carriers recombination and reactions at the electrolyte and electrode interfaces can be studied. In the present work, TAS has been carried out to elucidate the effect of hematite BL on primary charge dynamics at the NRs||electrolyte interface.

**Figure 7:**
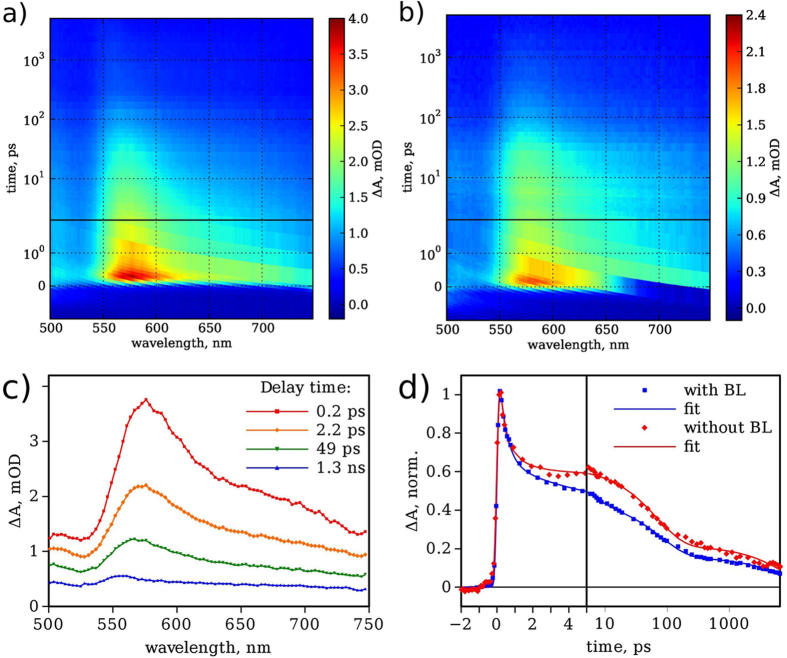
2D presentations of transient absorption measurements (time scale is linear till 2 ps and logarithmic at longer delays) of the electrode (**a**) FTO/hematite-BL/hematite-NRs and (**b**) FTO/hematite-NRs. (**c**) Time resolved transient absorption spectra at few delay times of the sample with hematite BL. (**d**) Normalized transient absorption decay at 580 nm for both photoelectrodes with and without BL. Excitation wavelength: 355 nm, applied bias: 1.23 V *vs* RHE.

Hematite shows a broad time-resolved spectrum right after the excitation, with a maximum centered at 580 nm (Fig. 7a,b), attributed to the absorption of photogenerated electrons and holes^52^,^53^, with an intensity slightly higher for the electrode featuring the BL. Two main contributions to the TAS come from photogenerated electrons and holes, which have broad but well separate absorption bands at 580 and 650 nm, respectively^53^. For both samples, with and without BL, the response is dominated by electrons (band at 580 nm) with barely visible contribution of holes (band at 650 nm). Another distinct feature of the samples is that the spectrum shape virtually does not change within few nanoseconds after excitation (Fig. 7c), but the signal intensity decreases gradually. This indicates a relatively high degree of losses due to carrier recombination and trapping.

Minor difference was observed between samples featuring the same architecture, as well as by measuring the same sample at different spots. The difference was within 20–30% range and can be attributed to sample inhomogeneity over the large area. Importantly, within this accuracy interval, there was virtually no difference between samples with and without BL as presented in Fig. 7d, which is the firm confirmation that BL has virtually no effect on dynamics of photogenerated carries in hematite and does not influence primary reactions at the NRs||electrolyte interface. The data presented in Fig. 7 were obtained with 1.23 V bias potential applied. Similar measurements were carried out with 0.8 and 1.6 V bias potentials, but the results were very similar and no distinguishable effect of BL was observed in the time interval up to 6 ns. Thus the transient absorption measurements support the conclusion that differences identified in photoelectrochemical responses are merely ascribable to the action as a physical insulator of FTO from electrolyte exerted by the hematite BL.

## Conclusions

We presented a simple and fast treatment able to enhance the photocurrent of hematite photoelectrodes. We demonstrated that a relevant increase of photocurrent delivered from a forest of hematite nanorods can be achieved through physical insulation of FTO from the electrolyte by a thin layer of the same material.

Results appear of interest as they were obtained with a little additional cost in terms of both materials and procedures: no complicated or expensive further processing, no doping or decoration with costly species, no production/dispersion of harmful materials. The compliance with similar criteria of green chemistry^54^ appears of particular value in technologies aiming at the use of natural, renewable and sustainable energy sources.

This study represents an example of how a rational, although simple, electrode engineering strategy holds the potential to significantly push forward the functional features of a material otherwise poorly performing: the path to fabricate efficient hematite-based photoelectrodes is still long but may consist of several significant steps like the one presented here.

## Methods

### Materials

Iron(III) Chloride (≥97%), sodium Nitrate (≥99%), ethanol (≥99.8%), acetylacetone (≥99%), Iron(III) tris(acetylacetonate) (Fe(C_5_H_7_O_2_)_3_) (≥97%) were purchased from Sigma Aldrich and used without further purification. Solutions for photoelectrochemical measurements were prepared from deionized water (ρ > 15 MΩ cm) and high purity chemicals (Sigma-Aldrich, puriss. p.a. ACS reagents). FTO glasses were purchased from XOP Glass (sheet resistance 15 Ω/cm^2^).

### Electrode preparation

Hematite BL is deposited on FTO glasses by spraying a 0.15 mM ethanolic solution of Fe(C_5_H_7_O_2_)_3_ (nozzle-to-sample distance: 25 cm, pressure of air carrier: 6 psi, 5 s spray followed by 30 s pause, total volume of sprayed precursor: 10 ml) on the substrate (T 400 °C). Hematite NRs are then grown by applying a standard hydrothermal procedure^10^, as it follows: a cleaned piece of FTO glass is put in a Teflon autoclave with a 0.15 M aqueous solution of FeCl_3_ containing 1M NaNO_3_ (pH is set at 1.5 using HCl) and heated in an oven at 95 °C for 24 h. After cooling down at room temperature, the sample is rinsed several times with distilled water and then dried at 60 °C in an oven. Electrodes are eventually annealed at 450 °C (1 h) to carry out the phase transition from β-FeOOH to α-Fe_2_O_3_.

### Characterization

*UV-Vis adsorption spectroscopy* UV-Vis spectra were recorded in a T80 PG Instruments in the range 1100–350 nm (resolution of 2 nm).

#### Raman spectroscopy

All Raman spectra were collected using a modular micro-Raman confocal system from Horiba equipped with a single monochromator (ihr320mst3) and a Peltier-cooled CCD camera. A solid state laser at 532 nm and 30 mW was used as the excitation source, along with interference filters on the laser lines and edge filters on the signal. The spectra were collected by a 100x objective operating with 1800 l/mm grating, with 60 s integration time, over a spectral range 150–1100 cm^−1^.

#### Atomic Force Microscopy (AFM)

AFM measurements were performed by means of a Bruker-Thermomicroscope CP-Research microscope working in non-contact mode at atmospheric pressure with a Si cantilever with 10 nm of radius.

#### Scanning electron microscopy (SEM)

SEM analysis was carried out by a field-emission LEO 1525 microscope, equipped with an In-Lens detector for secondary electron imaging.

#### Ultrafast Transient Absorption Spectroscopy

The TAS instrument and data handling procedures are described elsewhere^55^. In brief, the excitation wavelength was 355 nm and the time resolved transient absorption spectra were collected in the wavelength range 500–750 nm and at delay time up to 6.5 ns. Standard three electrode cell with Pt counter electrode, Ag/AgCl reference electrode and sample as working electrode was to bias the sample during the measurements. Electrolyte solution was 0.1 M NaOH + 0.2 M LiI in water and applied bias was 1.23 *vs* RHE.

#### Photoelectrochemical characterization

Experiments were performed in a pyrex electrochemical cell, with a three-electrode configuration, equipped with a quartz optical flat on the side. Experiments were recorded with a standard photoelectrochemical set up: 150 W Xe lamp and monochromator (Applied Photophysics); filters (Schott); light chopper (EG&G Brookdeal model 9479); potentiostat and function generator (Amel model 2053 and 568, respectively); lock-in amplifier (Perkin-Elmer model 5210). All potentials were measured versus a Hg/HgO, 1 M NaOH electrode and are referred in text and figures to the reversible hydrogen electrode (RHE):


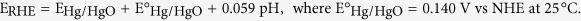


The pH value of 13.6 was attributed to 1.0 M NaOH solution^31^, whereas for the 0.1 M NaOH + 0.2 M LiI solution the approximate value pH = 13 was adopted^11^. White light power (up to about 1 sun) was estimated with a silicon reference cell. Monochromatic light power was measured with a calibrated photodiode (Macam Photometrics). Photocurrent-potential curves were obtained recording a linear sweep voltammetry (20 mV s^−1^) under white light illumination interrupted by the chopper (0.5 Hz). Photocurrent spectra were recorded feeding the reference signal from the chopper (2 Hz) and the output signal from the potentiostat to the lock-in amplifier. Signals were acquired in digital form after conversion by a multipurpose laboratory interface (Vernier). In some experiments the working electrode was illuminated through the electrolyte/electrode interface (EE experiments) or through the substrate/electrode interface (SE experiments), according to a literature terminology^11^.

## Additional Information

**How to cite this article**: Milan, R. *et al*. Compact hematite buffer layer as a promoter of nanorod photoanode performances. *Sci. Rep*. **6**, 35049; doi: 10.1038/srep35049 (2016).
